# Early Neurological Deterioration Despite Recanalization in Basilar Artery Occlusion Treated by Endovascular Therapy

**DOI:** 10.3389/fneur.2020.592003

**Published:** 2020-11-19

**Authors:** Xi Zhong, Xu Tong, Xuan Sun, Feng Gao, Dapeng Mo, Yilong Wang, Zhongrong Miao

**Affiliations:** ^1^Department of Neurology, Beijing Tiantan Hospital, Capital Medical University, Beijing, China; ^2^Department of Neurology, Peking University Shougang Hospital, Beijing, China; ^3^Department of Interventional Neuroradiology, Beijing Tiantan Hospital, Capital Medical University, Beijing, China; ^4^Department of Neurology, Tangshan Gongren Hospital, Hebei Medical University, Tangshan, China; ^5^China National Clinical Research Center for Neurological Diseases, Beijing, China; ^6^Center of Stroke, Beijing Institute for Brain Disorders, Beijing, China

**Keywords:** basilar artery, acute stroke, revascularization, prognostic factor, endovascular treatment

## Abstract

**Background:** We aim to identify the risk factors of early neurological deterioration (END) despite successful recanalization and explore the association of END with 90-day outcomes in acute basilar artery occlusion (BAO) treated by endovascular therapy (EVT).

**Methods:** A prospectively registered consecutive cohort of BAO patients with successful recanalization by EVT in a tertiary stroke center during a 6-year period was reviewed. END was defined as an National Institutes of Health Stroke Scale (NIHSS) score increase ≥4 points, or death, from baseline to 24 h after EVT. Multivariate logistic regression analysis was used to identify the risk factors of END. The 90-day outcomes between END and non-END groups were compared by Pearson's χ^2^ test.

**Results:** END was observed in 21 of 148 patients included in this study. Multivariate logistic regression analysis showed that patients with progressive or fluctuating symptoms had a higher risk of END than those with symptoms of acute attack (OR 5.52, 95% CI 1.73–17.63), and NIHSS score and puncture-to-recanalization time (PTR), as continuous variables, were also significantly associated with END. Using a generalized additive model with spline smoothing function, we observed a linear relationship between PTR (increased by 1 h) and END (OR 2.57, 95% CI 1.45–4.57), and a non-linear relationship between NIHSS score and END. Only when the NIHSS score was ≥23 points was it related to END (OR 0.7, 95% CI 0.6–0.9). In addition, patients with END had a lower proportion of 90-day favorable outcome (19.0 vs. 59.1%, *p* < 0.01) and higher mortality (33.3 vs. 13.4%, *p* = 0.048) than those with non-END.

**Conclusion:** Mode of stroke onset, NIHSS score, and PTR may help to identify BAO patients with a higher risk of END after EVT. Moreover, END may affect the 90-day outcomes of these patients.

## Introduction

Ischemic stroke due to acute basilar artery occlusion (BAO) accounts for about 1% of all ischemic strokes (1), but it is often associated with either severe neurological impairment or high mortality rate (2, 3). During the past several years, a number of new generation devices for endovascular revascularization have been introduced for BAO, and they seem to have more promising potential for achieving a high rate of revascularization than intravenous thrombolysis (4). However, poor clinical outcomes, despite successful revascularization, are still present in a large percentage of patients with acute BAO (5). The futile recanalization rate can be as much as 47% (6).

END is an index for the worsening of neurological symptoms, which could be caused by symptomatic intracerebral hemorrhage, malignant edema, and early recurrent stroke (7). According to different severity thresholds and the time scales between assessments, the incidence of END varies widely from study to study. The typical definition of END (8, 9) is an increase ≥4 points in the National Institutes of Health Stroke Scale (NIHSS) or death within the first 24 h after stroke, and END affects stroke patients' long-term outcomes (10–12).

Early studies mainly focused on END in patients who were treated with intravenous thrombolysis. Certain clinical as well as radiological variables were found to be strongly associated with END, which include the site of occlusion, initial hypoperfusion, infarct volume, NIHSS score on admission, and 24-h recanalization (7, 13). However, only a few studies have investigated the risk factors and their role in END prognosis in stroke patients after endovascular treatment (EVT), and no studies have investigated END in patients with acute BAO after successful recanalization.

The primary aim of this study was to identify the clinical factors associated with END and to clarify the effects of END on 3-month stroke outcomes.

## Methods

### Study Population

A prospectively registered cohort of patients with acute BAO (*n* = 187) treated by EVT (including stent-retriever thrombectomy and/or intra-arterial thrombolysis and/or emergency angioplasty) within 24 h of BAO onset at Beijing Tiantan Hospital between January 2012 and July 2018 was established. Subjects included only those with successful recanalization. Informed consent was obtained from all patients, or their legally authorized representatives, before EVT, and the study protocol was approved by the Ethics Committee of Beijing Tiantan Hospital. All data were anonymized, and the identities of patients were protected.

The time of BAO onset was described by the patients or witnesses; if unknown, it was considered to be the last time the patient was well. In patients with mild symptoms followed by sudden onset of decreased consciousness, the time of deterioration in clinical status was taken as the estimated time of BAO onset. Successful recanalization was defined as modified thrombolysis in cerebral infarction grade (mTICI score) ≥2b. Intravenous treatment with tissue plasminogen activator (tPA) before EVT was acceptable, in accordance with the current guidelines of the Chinese Society of Neurology/Chinese Stroke Society. Patients with a premorbid modified Rankin scale (mRS) score of >3 were excluded from this study.

### Demographic and Clinical Assessments

Patient's baseline and clinical characteristics (e.g., demographic data, NIHSS score, laboratory test results, neurovascular images, stroke subtypes, operative information, and perioperative management) and functional outcomes (e.g., mRS score) within 90 days were prospectively collected.

Neurological deficits of all patients were assessed by NIHSS score at baseline, 24 ± 2 h, 7 ± 1 days (or at discharge, whichever occurred first), and at any time of neurological deterioration. The mRS score was assessed at 7 ± 1 days (or at discharge, whichever occurred first) and 90 ± 7 days. Only neurologists trained and qualified to use the NIHSS and mRS recorded the scores.

Neurovascular image [CT plus CT angiography (CTA) and/or magnetic resonance (MR) plus MR angiography] were performed at baseline, within 24 h, and 7 days (or before discharge, whichever occurred first). Additional CT and/or MR were examined at any time of neurological deterioration. The imaging findings were interpreted by two independent trained radiologists blinded to the clinical data. A third experienced senior radiologist participated in the resolution of any disagreement.

### Outcome Measurement

Clinical functional outcomes according to the mRS were assessed at 90 days. A follow-up blinded to baseline information was carried out through telephone interviews by trained interviewers based on a standardized interview protocol. In this study, the outcome measures included functional independence, favorable outcomes, as well as death within 90 days after the procedure. Functional independence was defined as an mRS score ≤ 2, and favorable outcome as an mRS score ≤ 3, in accordance with the BASICS definition (3).

### Definition of END

With reference to earlier studies (8, 9), we modified the definition to define early neurological deterioration (END) after successful recanalization as an increase of ≥4 points in NIHSS score from baseline NIHSS score, or death, within 24 h after successful recanalization.

### Statistical Analysis

Study data were collected on standard forms, evaluated for completeness, and double keyed into an EpiData statistics data document. Baseline and outcome data were presented as mean (SD) and/or median [interquartile range (IQR)] for continuous variables. Frequency and/or proportion were used for categorical variables. In univariate analysis, independent-samples *t*-test and/or the non-parametric test (Mann–Whitney *U*-test) were used to compare means and/or medians, whereas Pearson's χ^2^ test or Fisher's exact test was used to compare frequencies and/or proportions. All factors achieving *p* < 0.1 on univariate analyses were entered into a multivariate logistic regression model to identify clinically relevant risk factors for END. Generalized additive models were used to visually assess the relationships between the continuous covariates (e.g., NIHSS, PTR) and the risk of END. We further applied a two-piecewise linear regression model to examine the threshold effect of the continuous covariates on END using a smoothing function. The threshold level (e.g., turning point) was determined using trial and error, including selecting of turning points along with a pre-defined interval and then choosing the turning point that gave the maximum model likelihood. We also conducted a log-likelihood ratio test comparing the one-line linear regression model with a two-piecewise linear model. All tests were two tailed and statistical significance was determined at *p* < 0.05. All statistical analyses were performed with the statistical software package R (http://www.R-project.org, The R Foundation) and Empowerstats (http://www.empowerstats.com; X&Y Solutions, Boston, MA, United States).

## Results

**Figure 1:**
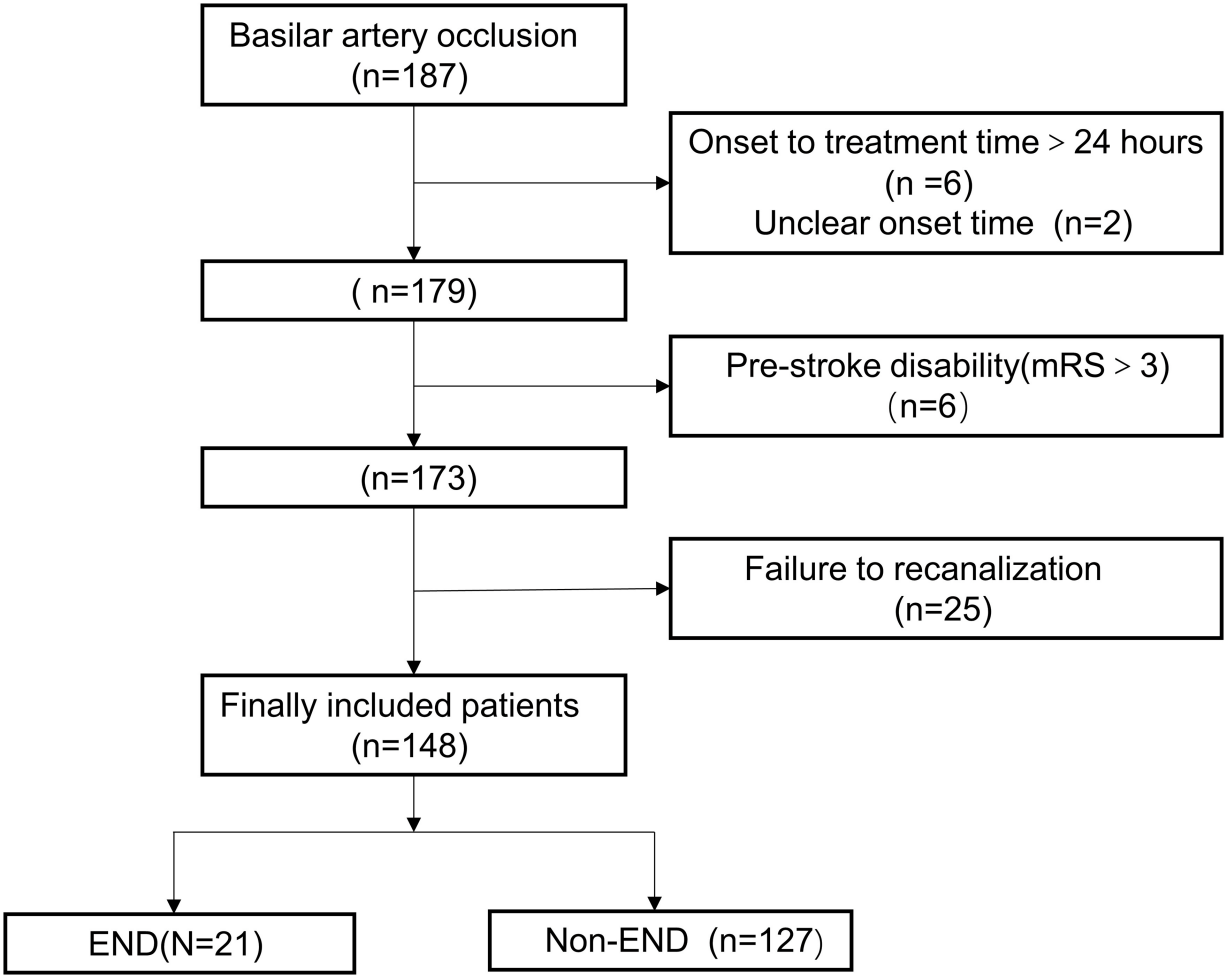
Flow chart outlining patient inclusion steps. END indicates early neurological deterioration; mRS, modified Rankin Scale.

Between January 2012 and July 2018, 187 consecutive patients with BAO underwent emergency EVT. Because of delayed treatment (>24 h) and unclear onset time, eight patients were excluded, and six patients with significant pre-stroke disability (mRS score >3) were excluded. In addition, 25 patients who did not achieve successful recanalization (mTICI score 2b−3) were also excluded. Finally, 148 eligible patients were included in the analysis (Figure 1). The median age (IQR) was 60 (54–67) years; 122 cases (82.4%) were male, and the median NIHSS score was 24 points (IQR, 10–34 points). A total of 118 cases (79.7%) were treated by mechanical thrombectomy with stent retrievers, and median onset-to-puncture time (OTP) was 6.5 h (IQR, 4.6–9.0 h). During a 90-day follow-up, the proportions of functional independence (mRS score ≤ 2), favorable outcome (mRS ≤ 3), and death were 39.9% (59 cases), 53.4% (79 cases), and 16.2% (24 cases), respectively.

END occurred in 21 patients (14.2%). The extent of deterioration was from 4 to 10 points in 11 patients, 11–20 points in six patients, ≥21 points in two patients, and two patients died within 24 h. Patients were divided into two groups based on those with END or not, and their clinical characteristics are summarized in Table 1.

**Table 1:** Characteristics of the patients with and without END.

**Characteristic**	**END (21)**	**Non-END (127)**	**P value**
Age, median (IQR), years	56 (49–65)	61 (54–67)	0.15
Male sex	18 (85.7)	104 (81.9)	0.91
**Vascular risk factors**			
Hypertension	19 (90.5)	85 (66.9)	0.03
Diabetes mellitus	4 (19.0)	33 (26.0)	0.50
Coronary heart disease	2 (9.5)	18 (14.2)	0.82
Previous stroke	4 (19.0)	27 (21.3)	>0.99
Current smoker	9 (42.9)	49 (38.6)	0.71
**Clinical characteristics**			
Mode of stroke onset			0.02
Abrupt	6 (28.6)	70 (55.1)	
Non-abrupt	15 (71.4)	57 (44.9)	
SBP, median (IQR), mmHg	160 (140–179)	160 (145–167)	0.92
NIHSS score, median (IQR)	15 (9–23)	28 (12–35)	0.03
White blood cells, × 10^9^/L	10.2 (3.8)	11.5 (3.9)	0.17
Glucose, median (IQR), mmol/L	7.4 (6.5–9.7)	8.2 (6.7–10.8)	0.23
Creatinine, median (IQR), μmol/L	63.3 (55.2–83.8)	64.9 (56.6–80.0)	0.81
pc-ASPECTS on DWI, median (IQR)	6 (5–8)	7 (5–8)	0.79
PMI on DWI, median (IQR)	3 (0–4)	2 (0–4)	0.83
Occlusion site			0.32
Proximal BA	1 (4.8)	20 (15.7)	
Middle and distal BA	20 (95.2)	107 (84.3)	
Tandem lesion	2 (9.5)	11 (14.0)	>0.99
Underlying ICAS	15 (71.4)	80 (63.0)	0.46
ASITN/SIR collateral score			0.81
0–1	10 (47.6)	53 (41.7)	
2	8 (38.1)	57 (44.9)	
3–4	3 (14.3)	17 (13.4)	
Stroke subtypes			0.11
Large artery arteriosclerosis	20 (95.2)	98 (77.2)	
Other etiology	1 (4.8)	29 (22.8)	
**Procedural features**			
Previous use of intravenous tPA	4 (19.0)	25 (19.7)	>0.99
General anesthesia	19 (90.5)	98 (77.2)	0.27
Use of stent retriever	19 (90.5)	99 (78.0)	0.30
Number of passes, median (IQR)	2 (1–2)	1 (1–2)	0.35
Intracranial stenting	13 (61.9)	54 (42.5)	0.10
Intra-arterial rt-PA or urokinase	3 (14.3)	26 (20.5)	0.41
Infusion of tirofiban	16 (76.2)	99 (78.0)	>0.99
Heparinization	10 (47.6)	47 (37.0)	0.36
Intracranial angioplasty			0.25
No	5 (23.8)	52 (40.9)	
Balloon alone	3 (14.3)	21 (16.5)	
Stenting	13 (61.9)	54 (42.5)	
Intra-procedural complications	7 (33.3)	18 (14.2)	0.05
Arterial perforation	3 (14.3)	4 (3.1)	
Arterial dissection	0 (0.0)	2 (1.6)	
Embolization in new territory	5 (23.8)	12 (9.4)	
Medical regimen			0.96
Tirofiban	14 (66.7)	81 (63.8)	
Dual or triple antiplatelet	5 (23.8)	32 (25.2)	
Mono-antiplatelet	2 (9.5)	14 (11.0)	
OTP, median (IQR) h	6.8 (5–13.5)	6.5 (4.5–9.0)	0.17
PTR, median (IQR) h	2.0 (1.5–2.5)	1.0 (1.0–2.0)	0.01

Values in parentheses are percentages, unless indicated otherwise.

IQR, interquartile range; mRS, modified Rankin scale; SBP, systolic blood pressure; NIHSS, National Institutes of Health Stroke Scale; pc-ASPECTS, posterior circulation Acute Stroke Prognosis Early CT Score; PMI, Pons–Midbrain Index; BA, basilar artery; ICAS, fixed stenosis degree >70% or >50% with distal blood flow impairment or evidence of repeated re-occlusion on intra-procedural digital subtraction angiography images (14); rt-PA, recombinant tissue plasminogen activator; OTP, onset-to-puncture time; PTR, puncture-to-recanalization time; DWI, diffusion-weighted imaging.

### Identification of Risk Factors for END

The pretreatment and procedural variables are presented in Table 1. In univariate analysis, patients with END had a higher percentage of hypertension (90.5 vs. 66.9%, *p* = 0.03), while no other vascular risk factors (sex distribution, diabetes mellitus, coronary heart disease, previous stroke, smoking status) were identified as risk factors for END. Compared with patients with abrupt symptoms, patients with non-abrupt symptoms were more likely to suffer END (*p* = 0.02) and the median NIHSS score in patients with END was lower (*p* = 0.03). Moreover, there was a significant difference (*p* = 0.01) in the medial PTR between patients with END (median, 2.0; IQR: 1.5–2.5) and those without END (median, 1.0; IQR: 1.0–2.0). The proportion of intraprocedural complications in the END group was higher than that in the non-END group, and the difference has a significant trend (*p* = 0.053). No findings in the neurovascular imaging factors (occlusion site, ASITN/SIR collateral status, tandem lesion, and stroke type) were associated with END, and operative information such as the type of anesthesia, use of stent retriever, as well as angioplasty method was also similar in both groups. There were no differences in presenting systolic blood pressure and laboratory test results between patients with and without END.

Multivariate logistic regression analysis adjusted for those relevant confounders with *p* ≤ 0.1 in univariate analysis. The results showed that mode of stroke onset (OR 5.52; 95% CI 1.73–17.63), PTR (*p* < 0.01), and baseline NIHSS score (*p* < 0.01) were independently associated with END (Table 2).

**Table 2:** Multiple logistic regression analysis for risk factors with early neurological deterioration after successful recanalization.

**Variable**	**OR (crude)**	**95% CI**	**P-value**	**OR (adjusted)**	**95% CI (adjusted)**	**P-value**
Mode of stroke onset
Abrupt				Reference		
Non-abrupt	3.07	1.12–8.42	0.03	5.52	1.73–17.63	<0.01
PTR (increased by 1 h)	2.12	1.30–3.46	<0.01	2.57	1.45–4.57	<0.01
NIHSS score (increased by 1 point)	0.96	0.92–0.99	0.03	0.93	0.88–0.97	<0.01

PTR, puncture-to-recanalization time; NIHSS, National Institutes of Health Stroke Scale; OR, odds ratio.

### Two-Piecewise Linear Regression Model for NIHSS Score and PTR

**Figure 2:**
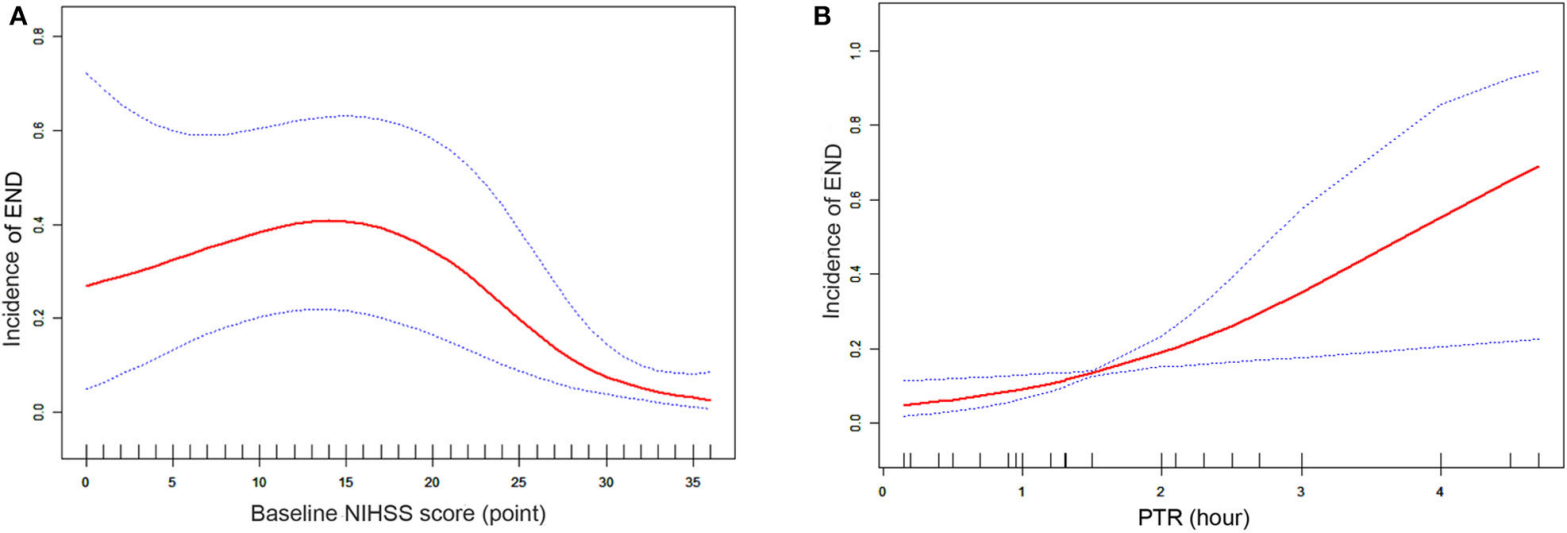
**(A)** Smooth curve fitting of 24-h END incidence and baseline NIHSS score (points). Red lines represent the spline plots of baseline NIHSS score and blue dotted lines represent the 95% CIs of the spline plots. Adjusted factors: mode of stroke onset and PTR. **(B)** Smooth curve fitting of 24-h END incidence and PTR (per hour). Red lines represent the spline plots of PTR and blue dotted lines represent the 95% CIs of the spline plots. Adjusted factors: mode of stroke onset and baseline NIHSS score.

Figure 2 shows smooth curves of the END by baseline NIHSS score and PTR. The smoothed curve showed that END was increasing linearly with PTR, whereas the relationship of END and NIHSS score was not simply linear, as a turning point could be seen at 23 (LLR test, *p* < 0.01), after adjusting for confounders including mode of stroke onset and PTR. In the group with NIHSS score >23, the odds ratio of END with age was 0.7 (95% CI 0.6–0.9; *p* < 0.01), but there was no significant relationship between END and NIHSS score when NIHSS score < 23 (*p* = 0.41; Table 3).

**Table 3:** Threshold effect analysis of NIHSS score on 24-hour END incidence using piecewise linear regression.

	**Crude OR (95% CI)**	***P*-value**	Adjusted OR^*^ (95% CI)	***P*-value**
NIHSS score < 23	1.1 (1.0–1.2)	0.10	1.0 (0.9–1.1)	0.41
NIHSS score ≥23	0.7 (0.6–0.9)	<0.01	0.7 (0.6–0.9)	<0.01

*Crude: no adjustment*.

* Adjusted for PTR and mode of stroke onset.

NIHSS, National Institutes of Health Stroke Scale; END, early neurological deterioration; OR, odds ratio.

### Post-Treatment Factors Associated With END

Post-treatment factors associated with END within 24 h and 7 days are shown in Table 4. There was a trend for significance for ICH within 24 h, higher in the END group (*p* = 0.08). However, END showed no association with extensive brainstem infarction and re-occlusion. END group had a significantly higher rate of symptomatic ICH within 24 h (<0.01) and 7 days (<0.01). According to the follow-up clinical outcomes within 24 h and within 7 days, patients who more frequently experienced END had hernia than those without END (*p* = 0.02). Among END patients, NIHSS score at 24 h (<0.01) and 7 days (<0.01) was significantly higher.

**Table 4:** Post-treatment factors associated with END.

**Characteristic**	**END (21)**	**Non-END (127)**	***P*-value**
**Follow-up imaging findings within 24 h and 7 days**			
Any ICH within 24 h	6/17 (35.3)	18/116 (15.5)	0.08
Symptomatic ICH within 24 h^a^	6/17 (35.3)	0/116 (0.0)	<0.01
Any ICH within 7 days	6/18 (33.3)	21/119 (17.6)	0.13
Symptomatic ICH within 7 days^a^	6/18 (33.3)	1/119 (0.8)	<0.01
Extensive brainstem infarction (PMI>3) within 24 h	3/17 (17.6)	30/116 (25.9)	0.56
Extensive brainstem infarction (PMI>3) within 7 days	6/18 (33.3)	37/119 (31.1)	0.85
Re-occlusion within 7 days^b^	1/21 (4.8)	12/127 (9.4)	0.69
**Follow-up clinical outcomes within 24 h and 7 days**			
Hernia within 7 days	5 (23.8)	7 (5.5)	0.02
NIHSS score at 24 h^c^	29 (18–33)	13 (6–26)	<0.01
NIHSS score at 7 days^d^, ^e^	28 (14–32)	10 (3–25)	<0.01

Values in parentheses are percentages, unless indicated otherwise. ICH, intracranial hemorrhage; PMI, Pons–Midbrain Index; NIHSS, National Institutes of Health Stroke Scale.

a According to the ECASS III definition.

b Assessment by CT angiography, MR angiography, digital subtraction angiography, or bedside transcranial Doppler.

c The NIHSS score was not available for 2 patients in the END group due to death.

d The NIHSS score was not available for 3 patients in the END group and 2 patients in the non-END group due to death.

e OR at discharge (whichever occurs first).

### Comparison of 90-Day Outcomes Between END and Non-END Groups

**Figure 3:**
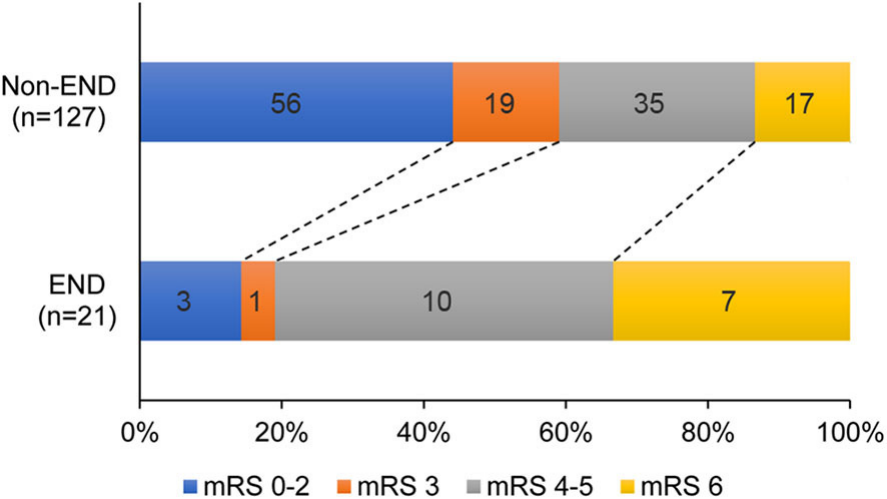
mRS scores at 90 days for patients with END vs. Non-END. END indicates early neurological deterioration; mRS, modified Rankin Scale.

Finally, the incidence rates of functional independence (14.3 vs. 44.1%, *p* = 0.01) and favorable outcome (19 vs. 59.1%, *p* = 0.001) at 3 months were much less prevalent in the END group than in the non-END group, and the mortality within 3 months was significantly increased in the END group (33.3 vs. 13.4%, *p* = 0.048; Figure 3).

## Discussion

The main findings of the study are as follows: (1) patients with progressive or fluctuating symptoms at stroke onset were more likely to suffer END; (2) “puncture to recanalization time” (PTR) is an independent risk factor associated with END in BAO; (3) we observed a non-linear relationship between NIHSS score and END.

Previous studies indicated that END was present in 10–38% of patients after IV rt-PA (15–18), and in 35.2–40.2% of patients after endovascular recanalization (18, 19). A recent meta-analysis showed that the pooled incidence of END, defined as a ≥4-point increase in NIHSS score between admission and at 24 h, was about 11.0% (20). END was seen in 13.5% of the patients in our study, the reason for the relatively low percentage possibly being we only included those who were successfully recanalized.

We found that the relationship between NIHSS score and END was not linear. The explanations for this finding may be that in our cohort, there were 76 patients with baseline NIHSS score ≥23 points, among which 60 patients (78.9%) had a score ≥30 points. As a result, the NIHSS score was hard to increase more than 4 points. This may be due to a ceiling effect, which prevented the high scores from increasing further (21). As we can see from our results, patients with higher NIHSS scores ≥23 points had a lower incidence of END, which was in contrast to some previous studies (11, 16) but consistent with several other studies (20, 22). Low NIHSS scores do not always represent mild ischemic symptoms (23) and mild stroke patients with symptomatic arterial occlusion were easier to develop END (24). Moreover, the BASICS study (25) suggested that prognostic power of 24- to 48-h NIHSS was higher than baseline NIHSS for 1-month poor outcome (area under the curve, 0.92 vs. 0.75) and mortality (area under the curve, 0.85 vs. 0.72). Therefore, END that reflects changes in neurological condition 24 h after surgery can serve as a better and more suitable surrogate of baseline NIHSS to predict long-term outcomes.

Time is one of the most important aspects of the management of acute ischemic stroke (AIS) and any delays in starting treatment will result in a worse outcome. PTR is an important time metric and some studies have shown that it is independently associated with 90-day outcomes (26, 27). Our findings suggested that PTR is an independent risk factor associated with END, which is in line with previous studies that showed an association between PTR and clinical outcomes (27, 28). PTR was significantly longer in the END group than in the non-END group; the possible reasons may be that (1) in our study, the rate of interventional complications in the END group was significantly higher, and Spiotta (27) has suggested that interventional complications are associated with increased procedure time. (2) According to our analysis, 11 patients (52.3%) in the END group experienced ≥2 times of stent pass, which indicates that vascular anatomy is highly sophisticated and thus the operation could be demanding. (3) The third possible explanation for this could be the characteristics of the thrombus. With larger or longer clots, it may require multiple attempts with different devices, which inherently involves a time penalty in the process (29, 30). (4) Patients with ICAS or tandem lesions tend to be refractory to thrombectomy treatment (31, 32), and rescue treatments such as angioplasty and/or stenting are often needed after thrombectomy to achieve good outcomes (14), which in turn prolonged the procedure time. (5) Finally, in some cases, some patients had to change from local anesthesia to general anesthesia during the procedure time, which also increased PTR.

The relatively low frequency of END in patients with an abrupt mode of onset is not an unexpected finding. It is easy to conceive that a cardiac embolus leads to acute occlusion of a major cerebral artery, giving rise to a sudden onset of neurological deficits. Furthermore, significant stenoses in intracranial arteries were found to be more frequent in progressive ischemic strokes in a previous study (33). Several studies have shown that intracranial atherosclerotic stenosis (ICAS) displays a unique risk factor profile as well as technical challenges for endovascular reperfusion therapy (34) and it has relatively poor functional outcomes compared with embolic occlusion (35). Furthermore, the onset time is less precise in patients with fluctuating symptoms as patients with acute symptoms, so that the delay is underestimated.

Our study demonstrated that END was associated with symptomatic hemorrhage and hernia within 7 days and was also associated with poor outcome at 3 months. Similar findings have been consistently reported in previous studies (10, 18). There is no significant correlation between extensive brainstem infarction and END. The possible reason may that we adapted two imaging techniques (CT/MRI), which led to the heterogeneity of PMI scores.

The impact of END on long-term outcome warrants standardized treatment protocols to reduce procedural time in endovascular treatment, thus possibly lowering the risk of END.

Our study has some limitations. First, this study is a retrospective analysis of a database in a single center, and the proportion of general anesthesia employed in this study is relatively higher, so, although it is prospectively maintained, its generalizability may be limited. Second, our study used the most commonly used definition of NIHSS score increase ≥4 after vascular recanalization as END, and this cannot completely reflect the severity and deterioration of posterior circulation infarction, although there is no alternative superior method. Third, due to incomplete imaging data, we were unable to classify and analyze the etiology of END. Finally, the sample size of the END group was relatively small, which may have resulted in low test efficiency, and thus the overlooking of some risk factors.

## Summary

In conclusion, our study highlights the importance of PTR in the interventional management of acute BAO. In contrast to many non-modifiable predictors of outcome in stroke, such delays can be greatly improved with appropriate systemic changes. Time guidelines and standardized treatment protocols should aim to reduce procedural times in endovascular treatment, thereby possibly improving outcomes among patients with acute ischemic stroke.

## Data Availability Statement

The raw data supporting the conclusions of this article will be made available by the authors, without undue reservation.

## Ethics Statement

The studies involving human participants were reviewed and approved by the Ethics Committee of Beijing Tiantan Hospital. The patients/participants provided their written informed consent to participate in this study.

## Author Contributions

XZ and XT: conceptualization, methodology, data analysis, and article drafting. XS, FG, and DM: data curation and coordination. YW and ZM: supervision and obtaining funding. All authors contributed to the article and approved the submitted version.

## Conflict of Interest

The authors declare that the research was conducted in the absence of any commercial or financial relationships that could be construed as a potential conflict of interest.
